# Scopolamine Induces Deficits in Spontaneous Object-Location Recognition and Fear-Learning in Marmoset Monkeys

**DOI:** 10.3389/fphar.2017.00395

**Published:** 2017-06-21

**Authors:** Jonathan L. Melamed, Fernando M. de Jesus, Rafael S. Maior, Marilia Barros

**Affiliations:** ^1^Department of Pharmaceutical Sciences, School of Health Sciences, University of BrasiliaBrasilia, Brazil; ^2^Primate Center and Department of Physiological Sciences, Institute of Biology, University of BrasiliaBrasilia, Brazil

**Keywords:** marmoset, object location, recognition, snake, fear memory, scopolamine

## Abstract

The non-selective muscarinic receptor antagonist scopolamine (SCP) induces memory deficits in both animals and humans. However, few studies have assessed the effects of amnesic agents on memory functions of marmosets – a small-bodied neotropical primate that is becoming increasingly used as a translational model for several neuropathologies. Here we assessed the effects of an acute SCP administration (0.03 mg/kg, sc) on the behavior of adult marmoset monkeys in two tasks. In the spontaneous object-location (SOL) recognition task, two identical neutral stimuli were explored on the sample trial, after which preferential exploration of the displaced versus the stationary object was analyzed on the test trial. In the fear-motivated behavior (FMB) procedure, the same subjects were submitted to an initial baseline trial, followed by an exposure period to a snake model and lastly a post-exposure trial. All trials and inter-trial intervals lasted 10 min for both tests. Results showed that on the SOL test trial, the saline group explored the displaced object significantly longer than its identical stationary counterpart, whereas SCP-treated marmosets explored both objects equivalently. In the FMB test, the saline group – but not the SCP-treated animals – spent significantly less time where the stimulus had been specifically encountered and more time being vigilant of their surroundings, compared to pre-exposure levels. Drug-related effects on general activity, overall exploration (SOL task) and behavioral response to the aversive stimulus (FMB task) were not observed. SCP thus impaired the marmosets’ short-term ability to detect changes associated with the spatial location of ethologically irrelevant (SOL task) and relevant stimuli (FMB task). Similar results have been reported in other animal species. Marmosets may thus help reduce the translational gap between pre-clinical studies and memory-associated human pathologies.

## Introduction

Over the years central cholinergic signaling has become increasingly implicated in different learning and memory processes (Hasselmo and Sarter, 2011). In fact, the loss of specific basal forebrain cholinergic input to the cortex is one of the pathogenic hallmarks of Alzheimer’s dementia (Bartus, 2000), with a concomitant decline in cortical choline acetyltransferase (ChAT) activity also being correlated with cognitive dysfunction in different human pathologies (e.g., dementias, Parkinson disease, brain damage; Candy et al., 1983). In rodents and non-human primates (NHPs), the use of excitotoxic (e.g., *rodents*: Baxter and Bucci, 2013; *marmosets*: Ridley et al., 1986; *macaques*: Aigner et al., 1991a) and more specific immunotoxic lesions (e.g., *rodents*: Easton et al., 2011; *marmosets*: Ridley et al., 1999; *macaques*: Turchi et al., 2005) of basal forebrain cholinergic projections to the cortex disrupted several learning and memory processes. When using this approach, the degree of the impairment can vary significantly according to the specificity and extent of the lesion and the type of cognitive task being assessed, with the possible involvement of non-cholinergic afferents. However, recent optogenetic-based studies have provided compelling evidence in mice for a causal role of basal forebrain cholinergic activity during visual discrimination tasks (Pinto et al., 2013).

The acetylcholine (ACh) muscarinic receptor blocker scopolamine (SCP) is also reported to disrupt memory processes in humans (Ebert and Kirch, 1998), whereas restoration of transmitter functioning reverses this effect (i.e., cholinesterase inhibitors; Roman and Rogers, 2004). There is also now substantial evidence for its participation in memory-related task performance of both rodents and NHPs (Klinkenberg and Blokland, 2010; Robinson et al., 2011; Baxter and Bucci, 2013), yielding similar results as those seen in lesion studies (Robinson et al., 2011). In fact, SCP has become a frequently used pre-clinical pharmacological tool to assess memory (dys)function (Klinkenberg and Blokland, 2010).

Scopolamine administration, for example, can consistently impair NHPs in delayed nonmatching-to-sample tasks (DNMS) of visual recognition memory (e.g., Ridley et al., 1984b,a; Aigner et al., 1991b). Although this task exploits their spontaneous preference for novelty over familiarity, it requires pre-training the monkey to learn response-reward associations and the nonmatching to sample rule. Rodents, on the other hand, are typically assessed in a simpler procedure requiring no prior training or response reinforcement – the one-trial spontaneous object recognition task and its several close variations (Dere et al., 2007). Granted that this procedure also exploits their novelty preference, its basis is the spontaneous explorative behavior displayed during a choice trial that occurs after an initial familiarization period. When treated with SCP, rodents become unable to recognize familiar objects (reviewed in Dere et al., 2007) or their associated spatial locations (Murai et al., 2007; Pitsikas, 2007; Barker and Warburton, 2009; Schäble et al., 2012). Originally tested in rats by Ennaceur and Delacour (1988), spontaneous recognition tasks have since been extended to other animals (e.g., *mice*: Dere et al., 2005; *dogs*: Callahan et al., 2000; *pigs*: Kornum et al., 2007), but to the best of our knowledge still remain to be assessed in NHPs. The ability to recognize whether an object has been encountered in the past is an important element of our declarative memory and a function that becomes impaired, for example, in patients with Alzheimer’s disease (Purdy et al., 2002) or who have sustained brain injury (Reed and Squire, 1997).

Cholinergic signaling also seems to play an important modulatory role on fear memories, a type of associative learning that has a high adaptive function against real and potential threats (Tinsley et al., 2004). For instance, during contextual conditioning, a neutral spatial location will come to evoke fear-related behaviors after being associated with an inherently fearful stimulus (Maren et al., 2013). In rodents, fear-conditioned stimuli increased central ACh release (Acquas et al., 1996), whereas SCP-treated animals performed poorly in conditioning tasks (reviewed in Robinson et al., 2011; Wilson and Fadel, 2017). Research on fear memory in NHPs, however, has focused mainly on elucidating the neuronal circuits involved in specific behavioral tasks (*fear-potentiated startle*: Antoniadis et al., 2009; *passive avoidance*: Machado et al., 2009; *cue-conditioning*: Agustín-Pavón et al., 2012). As fear memory processes seem to be altered in several psychopathologies (i.e., posttraumatic stress disorder and schizophrenia; Maren et al., 2013), as well as Alzheimer’s disease and other related dementias (Hoefer et al., 2008), new pharmacological-based studies in NHPs may contribute to our current understanding on the neurochemical aspects of learned fear.

The present experiments were thus designed to assess – in both the presence and absence of an acute SCP administration – the behavioral response of adult marmoset monkeys in a spatial recognition memory task and a fear-motivated learning procedure. The marmoset is a small-bodied, diurnal and arboreal neotropical primate. Compared to most NHPs they have a rapid reproductive turnover, shorter life-span, are easily captured and handled, readily adapt to captive conditions and have lower husbandry costs (reviewed in Barros and Tomaz, 2002). These characteristics, along with the recent sequencing of the common marmoset’s genome (*Callithrix jacchus*; reviewed in Ward and Vallender, 2012) and development of transgenic individuals (Sasaki et al., 2009) are making these simians an increasingly used translational model of several neuropathologies (‘t Hart et al., 2012). In fact, their small lissencephalic brains still retain a large brain-to-body ratio, a well-defined temporal lobe, functional divisions and connectivity of cortical areas, and structure-specific adult neurogenesis similar to those of other anthropoids (e.g., macaques; Stephan et al., 1980; Newman et al., 2009; Burman et al., 2011; Marlatt et al., 2011). Normal adults display the same cytochemical organization of basal forebrain cholinergic neurons of other NHPs and humans, which differs significantly from that of rats (Geula et al., 1993; Wu et al., 2000). Aged marmosets also develop cortical deposits of the beta-amyloid protein typically seen in Alzheimer’s dementia patients (Maclean et al., 2000; Geula et al., 2002). Marmosets are capable of performing a variety of memory-related tasks, yet only a few studies have assessed the effects of amnesic agents in marmosets (Ridley et al., 1984b,a; Carey et al., 1992; Harder et al., 1998; Spinelli et al., 2006).

In the first experiment, we used the murine-based one-trial spontaneous object-location (SOL) task (Ennaceur et al., 1997), while in the second experiment contextual fear learning was induced by a snake-related stimulus. Marmosets are highly visually oriented (Forster, 1995), readily attend to spatial cues in their environment (Gaudio and Snowdon, 2008) and react fearfully in response to snakes and related stimuli (Barros et al., 2002).

## Materials and Methods

### Ethics Statement

This study was carried out in accordance with the recommendations of the Brazilian regulations for the scientific use of laboratory animals (Lei Arouca 11.794/2008), as well as the CONCEA/Brazil and NIH/USA guidelines for care and use of laboratory animals. All the procedures herein were approved by the Animal Ethics Committee of the University of Brasilia (no. 33002/2013).

### Subjects and Housing Conditions

Nine adult black tufted-ear marmosets were used (*Callithrix penicillata*; 5 males and 4 females), weighing 344 ± 16 g (mean ± SEM; range: 285–460 g) at the beginning of the study. Although the females’ estrous cycle was not controlled, none were currently breeding or recently had infants. All subjects were pair-housed at the Primate Center of the University of Brasilia under natural light, temperature and humidity conditions in standard home-cages of a same colony room. Not all cage-mates were included in the present study due to other ongoing experiments. The colony room consisted of two parallel rows of 12 cages each (2 m × 1 m × 2 m; W × L × H), separated by a common wire-mesh enclosed central corridor. A roof covered this central corridor and two-thirds of each home-cage. These were provided with a nest-box, ropes, wood perches, a feeding tray for fresh food and a PVC tube for dry chow. Fresh food was provided daily at 07:30 h, consisting of a mixture of pieces of fruits and vegetables. Boiled eggs, nuts and/or cooked chicken breast were given several times a week, also at 07:30 h. Unconsumed items were removed at 17:30 h. Water and chow were available ad libitum. Housing and maintenance conditions complied with the regulations of the Brazilian Institute of Environment and Renewable Natural Resources (IBAMA).

### Apparatus and Experimental Set-up

**FIGURE 1:**
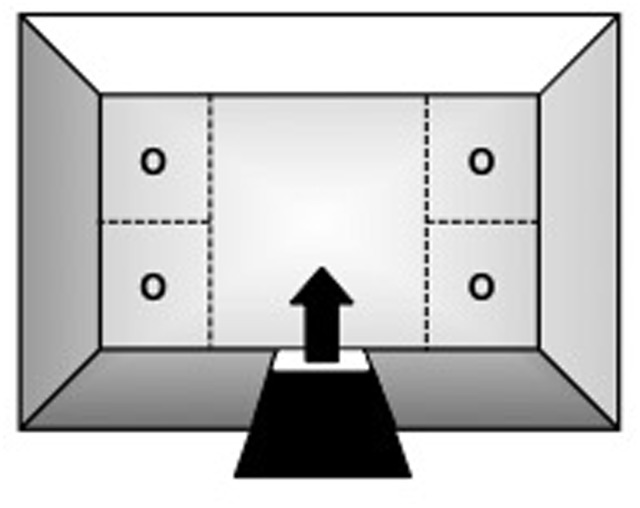
Top-view schematic representation of the marmoset open field apparatus, indicating the subjects’ entry/exit point (arrow) via the guillotine-type door, the four corner sections where stimuli could be placed during specific trials of the procedure (O), and the larger central zone.

Testing was conducted in a rectangular open-field (OF) arena (**Figure 1**: 130 cm × 75 cm × 40 cm; W × L × H) suspended 1 m from the floor. Three of its walls were made of aluminum, whereas the fourth was of 4 mm transparent glass. The top consisted of the same glass material and the bottom was made of 2.5 cm^2^ wire-mesh. A guillotine-type door on one of the aluminum walls served as the subjects’ entry/exit point. With the exception of the glass wall and top, the apparatus was painted white to enhance video-tracking. It was also divided into five quadrants (**Figure 1**): four corner sections of equal dimensions (32.5 cm × 37.5 cm each; W × L) and a larger central zone (65 cm × 75 cm; W × L).

The OF arena was set-up in a test-room located approximately 50 m from the colony facility. The marmosets were transported to and from the test-room in an aluminum transportation cage (35 cm × 20 cm × 23 cm; W × L × H) that attached directly to the arena’s door. The apparatus was monitored via a closed-circuit system with two digital cameras (model C920, Logitech, Brazil): one was mounted 1.5 m above the arena and the other was placed 1.5 m in front of its glass wall. Both cameras were connected to a same laptop located in an observation-room adjacent to the test-room. Visual spatial cues were provided by various extra-field items in the test-room.

### Drug

Scopolamine hydrobromide (SCP; 0.03 mg/kg; Sigma–Aldrich, United States) was dissolved in phosphate-buffered saline, the latter also being used as vehicle control (SAL). Both substances were injected subcutaneously in a volume of 1.0 mL/kg. The dose and injection-test interval (see Procedure below) were based on previous reports using systemic administrations in marmosets, whereby an inverted U-shaped function was verified (Ridley et al., 1984b: 0.03–1.0 mg/kg; Ridley et al., 1984a: 0.03–0.06 mg/kg; Carey et al., 1992: 0.01–0.04 mg/kg; Harder et al., 1998: 0.06 mg/kg; Spinelli et al., 2006: 0.01–0.06 mg/kg). In these studies, lower doses ranging from 0.02 to 0.06 mg/kg impaired performance in the object discrimination, position discrimination, visuospatial conditional, five-choice serial reaction time and concurrent delayed match-to-position tasks. On the other hand, SCP given at 0.05 or 1.0 mg/kg induced behavioral agitation in marmosets and thus may confound its specific memory effects at higher doses. Based on these studies, we chose to use 0.03 mg/kg as it may selectively disrupt memory, but not other behaviors.

### Procedure

All subjects were initially submitted, during three consecutive days, to a daily habituation session that mimicked the general procedure of the subsequent behavioral tasks (see below). Accordingly, each habituation session consisted of an initial 10 min trial, followed by a 10 min inter-trial interval and then a second 10 min trial. The marmoset was given access to the OF apparatus during these two trials of each session, while during the inter-trial interval they were placed in a different holding arena (60 cm × 60 cm × 40 cm; W × L × H) located in the same test-room. The marmosets were transferred between these locations using the transportation cage that attached directly to either arena. The three daily habituation sessions were to familiarize the marmosets with the apparatus and general testing procedure, and thereby no treatment was given and the OF remained empty.

The subjects were then randomly assigned to an experimental group (SCP: *n* = 5 or SAL vehicle: *n* = 4) and individually submitted to the same behavioral tasks described below. On both tasks, the specific location of the objects within the apparatus varied randomly between subjects. The apparatus and objects used were also thoroughly cleaned with a 70% ethanol solution after every trial. All trials were held between 14:00 and 17:00 h.

#### Spontaneous Object-Location Recognition Task

Based on the murine SOL task (Ennaceur et al., 1997), the marmosets were submitted to a two-trial procedure consisting of an initial 10 min sample trial that was followed, after a 10 min inter-trial interval, by a 10 min test trial. On the sample trial, two identical copies of a small stainless steel bowl (9 cm diameter x 5 cm height) were randomly placed at the center of different corner quadrants of the apparatus and the marmoset was allowed to freely explore the entire arena for 10 min. The objects had not been previously seen by the marmosets, had no apparent ethological significance and could not be displaced by the subjects. After the 10 min retention interval, held in the separate holding arena described above, the subject was again released in the OF for the 10 min test trial. On this trial, two identical copies of the same stainless steel bowl were placed in the arena: one in the same location it had been during the preceding sample trial (stationary object) and the other one in a new position randomly chosen between the previously two unused corner sections (displaced object). The marmoset was again allowed to freely explore the entire arena for 10 min and then returned to its home-cage.

Each subject received its respective treatment 20 min before the start of the SOL task. Systemically administered SCP exerts significant effects on central neuronal function 30 min post-injection (Ebert et al., 2001) and only pre-training SCP treatment has been found to impair SOL recognition memory in rodents (Barker and Warburton, 2009).

#### Fear-Motivated Behavior (FMB) Test

After a 2-week interval, the same two groups of marmosets were submitted to a three-trial procedure. First, a 10 min baseline pre-exposure trial was held in the OF arena in the absence of any stimulus. After a 10 min inter-trial interval held in the same holding arena, the subject was again released in the apparatus for a 10 min snake exposure trial. For this, a coiled and motionless red-black-white rubber snake model (120 cm long× 2 cm girth) was placed in one of the corner quadrants of the apparatus. As a general preference for any of the corner sections of the OF arena was not observed during the initial baseline trial, the snake model was randomly placed in any one of these locations. The subjects were all snake-naive and unable to displaced this aversive stimulus, which in turn could be seen from any point in the arena. After a second 10 min inter-trial interval held in the holding arena, the marmoset was placed for the third time in the OF apparatus for a 10 min post-exposure trial, in the absence of any stimulus, and thereafter returned to its home-cage.

Each subject received its respective treatment immediately before the start of the initial baseline trial. A snake model was used as an aversive stimulus since NHPs invariably regard them as a potential threatening stimulus (Isbell, 2006). Both feral (Teixeira et al., 2016) and captive marmosets (Barros et al., 2002) promptly react to snakes and related stimuli.

### Behavioral Analyses

We used the AnyMaze software (Stoelting Co., United States) to record and analyze the marmosets’ behavioral response during each experimental trial. Via the top-view camera, the software automatically tracked the animals’ total distance traveled and the time spent in each quadrant of the apparatus. In addition, using the same program and the side-view camera, an experienced observer with a 95% intra-rater reliability manually scored the time that the marmoset spent: (1) exploring each object during the SOL task; (2) visually inspecting the snake stimulus during the FMB task; (3) emitting the alarm/mobbing-associated *tsik-tsik* calls during the FMB task; and (4) being vigilant during the FMB task. Exploration in the SOL task was defined as physical contact with one of the objects using the hands, feet, nose, mouth, or tongue, as well as all episodes of head cocks (side-to-side head movements), direct gazes (fast orientation of the eyes and head toward the object) and visual monitoring the object (continuous slow sweeping movements of the head). Visual inspection of the snake model included head cocks, direct gazes and visual monitoring of this object, whereas vigilance was defined as visual monitoring directed at the environment. Marmosets are highly visually oriented in their response to surrounding stimuli (Forster, 1995).

For the SOL task, all subjects were included in the analyses below as they met our pre-established criterion of exploring each object for at least 5 s during the sample trial. Recognition memory was operationally defined as a higher exploration of the displaced versus stationary object on the test trial (e.g., Dere et al., 2007), considering that captive marmosets preferably explore novel items in their environment (Forster, 1995). However, to account for individual variations in overall exploration levels, the following discrimination ratio was calculated based on Ennaceur et al. (1997): [time spent exploring the displaced object – time spent exploring the stationary object]/[time spent exploring both objects]. A ratio of ≈ 0.0 indicates that the two objects were explored almost equally (chance level), whereas a ratio >0.0 demonstrates that the displaced object was explored more than the stationary item. For the FMB procedure, we assessed the subjects’ fear-induced place-avoidance response by comparing the time spent in the snake-paired section of the OF arena before and after the exposure trial (pre- x post-exposure trial).

### Statistical Analyses

Data from males and females in each group were pooled together as the small sample size precluded any meaningful gender comparisons. For the SOL task, the time spent exploring the displaced versus stationary object on the test trial, as well as total exploration and distance traveled on the sample versus test trial, were analyzed using a mixed-design two-way analysis of variance (ANOVA), with ‘treatment group’ as the independent factor and ‘object’/‘trial’ as the repeated measure variable. In addition, the discrimination ratios were compared to (zero value) chance-level performance via one-sample *t*-test. For the FMB task, an independent *t*-test was used for between-group comparisons regarding the visual inspection of the snake model and *tsik–tsik* vocalizations during the exposure trial. Dwell time in snake-paired quadrant, vigilance, distance traveled, and time spent in each corner section of the OF arena were analyzed via a mixed-design two-way ANOVA, with ‘treatment group’ as the independent factor and ‘trial’/’section’ as the repeated measure variable. Whenever significant effects were obtained in the ANOVA analyses, subsequent comparisons were performed using Tukey’s test. Significance level for all tests was set at *p* ≤ 0.05.

## Results

**FIGURE 2:**
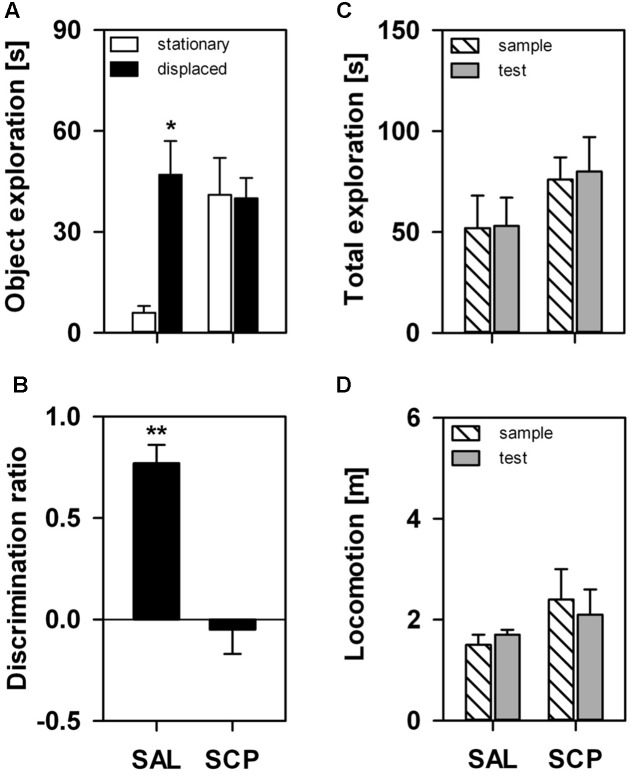
Marmosets’ performance on the one-trial *Spontaneous Object-Location (SOL)* Recognition Task. An acute saline (SAL; *n* = 4) or scopolamine (SCP, 0.03 mg/kg SCP; *n* = 5) administration was given subcutaneously 20 min before the task. The sample and test trials lasted 10 min and were held at 10 min intervals. **(A)** Time spent in seconds exploring the stationary and displaced objects on the test trial; **(B)** Object discrimination ratio calculated for the test trial; **(C)** Time spent in seconds exploring both objects on the sample and test trials; and **(D)** Total distance traveled in meters during the sample and test trials. All data are represented as mean + SEM. ^∗^*p* < 0.05 vs. the stationary object of the SAL group, ^∗∗^*p* < 0.05 vs. zero-value chance level in the SAL group.

On the SOL test trial, the displaced object was explored for a significantly longer time than the stationary one, albeit only in the SAL-treated group (object effect: *F*_1,7_ = 5.12, *p* = 0.05; treatment effect: *F*_1,7_ = 2.41, *p* = 0.17; interaction: *F*_1,7_ = 5.98, *p* = 0.04; **Figure 2A**). The COC-treated animals explored both objects equivalently on the test trial. The SAL-treated marmosets explored the displaced object significantly above chance level on the test trial (*t*_3_ = 8.97, *p* = 0.003), while the SCP group did not (*t*_4_ = –0.47, *p* = 0.67; **Figure 2B**). This response was not significantly influenced by either a trial or treatment effect on the marmosets’ overall exploration of the objects or by the level of locomotion, as both parameters remained constant between the sample and test trials (*object exploration* – trial effect: *F*_1,7_ = 0.07, *p* = 0.80; treatment effect: *F*_1,7_ = 3.44, *p* = 0.11; interaction: *F*_1,7_ = 0.001, *p* = 0.97; *distance traveled* – trial effect: *F*_1,7_ = 0.11, *p* = 0.75; treatment effect: *F*_1,7_ = 0.86, *p* = 0.39; interaction: *F*_1,7_ = 0.30, *p* = 0.60; **Figures 2C,D**).

**FIGURE 3:**
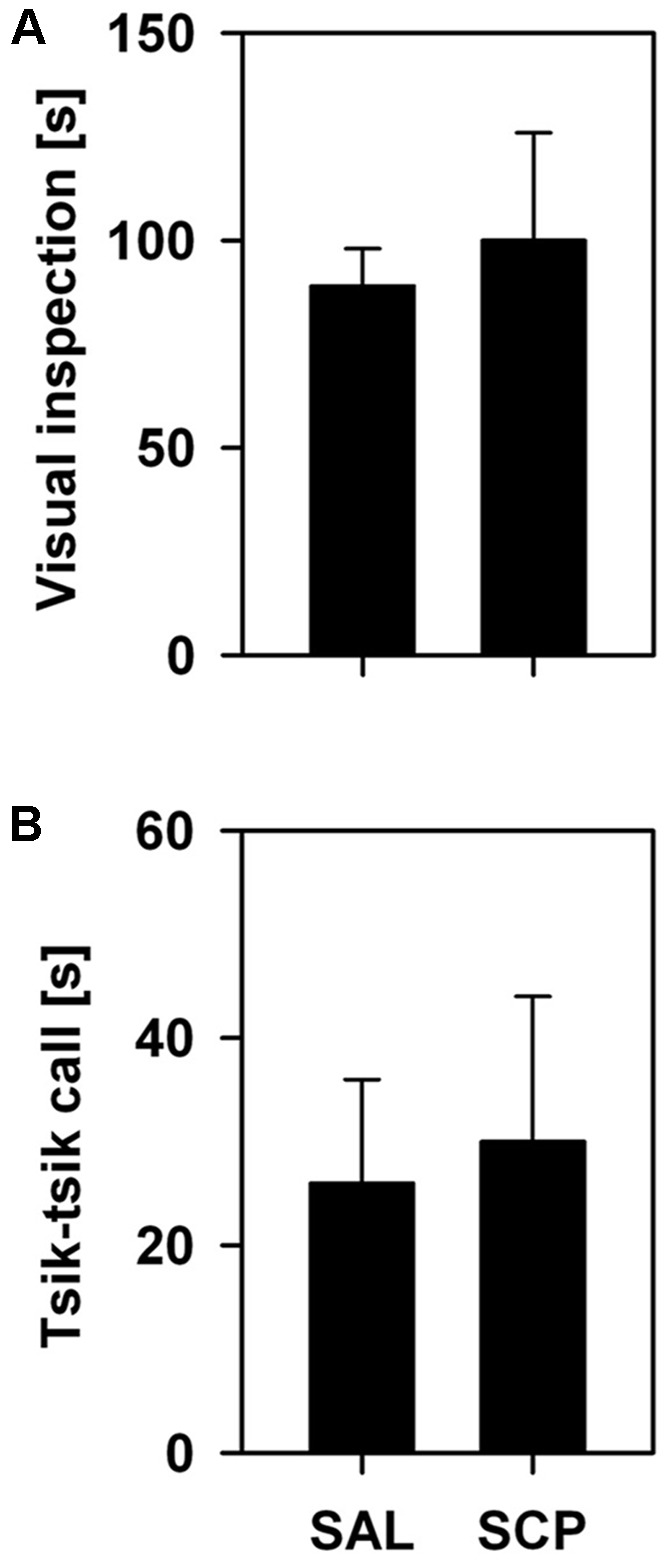
Mean time (+SEM; in seconds) spent visually inspecting the snake model **(A)** and emitting *tsik–tsik* alarm vocalizations **(B)** by the saline (SAL; *n* = 4) and SCP (0.03 mg/kg SCP; *n* = 5) treated marmosets during the 10 min snake-exposure trial of the Fear-Motivated Behavior (FMB) procedure.

**FIGURE 4:**
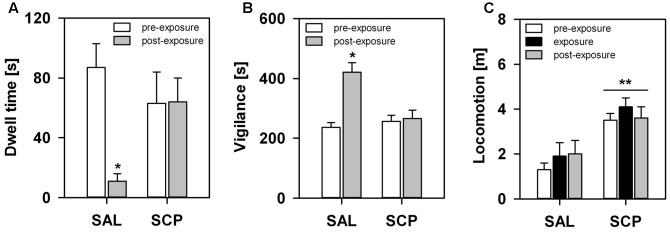
Marmosets’ behavioral response in the FMB procedure. An acute saline (SAL; *n* = 4) or SCP (0.03 mg/kg SCP; *n* = 5) administration was given subcutaneous immediately before the pre-exposure trial. All trials lasted 10 min and were held at 10 min intervals. Time spent in seconds **(A)** in the snake-paired quadrant of the open-field apparatus and **(B)** vigilant of the surroundings, before (pre-exposure trial) and after (post-exposure trial) being exposed to the snake model. **(C)** Total distance traveled in meters during each of the three trials of the procedure. All data are represented as mean + SEM. ^∗^*p* < 0.05 vs. the pre-exposure trial of the SAL group, ^∗∗^*p* < 0.05 SCP vs. SAL group.

During the initial baseline trial of the FMB task, held in the absence of the snake stimulus, all marmosets spent a comparable amount of time in the four corner quadrants of the OF arena (*SAL group* – section 1: 72 ± 18, section 2: 77 ± 19, section 3: 71 ± 13, section 4: 80 ± 9; *SCP group* – section 1: 69 ± 19, section 2: 78 ± 21, section 3: 74 ± 22, section 4: 78 ± 18; mean ± SEM in seconds; quadrant effect: *F*_3,21_ = 0.09, *p* = 0.85; treatment effect: *F*_1,7_ = 0.01, *p* = 0.99; interaction: *F*_3,21_ = 0.02, *p* = 0.97). On the conditioning trial, now in the presence of the snake model, the two groups also spent a similar amount of time visually inspecting the aversive stimulus (*t*_7_ = –0.36, *p* = 0.73; **Figure 3A**) and emitting *tsik-tsik* alarm calls (*t*_7_ = –0.22, *p* = 0.84; **Figure 3B**). However, after being confronted with the aversive stimulus, the SAL-treated marmosets spent significantly less time in the snake-paired quadrant of the OF apparatus compared to the levels seen prior to its exposure (baseline × test trial), whereas the SCP group spent a similar amount of time in this section on both trials (trial effect: *F*_1,7_ = 5.80, *p* = 0.04; treatment effect: *F*_1,7_ = 0.75, *p* = 0.41; interaction: *F*_1,7_ = 5.92, *p* = 0.04; **Figure 4A**). The SAL-treated marmosets were also found to be significantly more vigilant following the snake exposure, relative to the pre-confrontation levels of the baseline trial. Vigilance recorded in the SCP group remained unaltered between the baseline and test trials (trial effect: *F*_1,7_ = 17.68, *p* = 0.004; treatment effect: *F*_1,7_ = 4.07, *p* = 0.08; interaction: *F*_1,7_ = 14.05, *p* = 0.007; **Figure 4B**). Finally, the total distance traveled by the SCP-treated animals was significantly greater than that of the SAL group (*F*_1,7_ = 21.94, *p* = 0.002), however no between-trial effect (*F*_2,14_ = 1.28, *p* = 0.30) or trial-treatment interaction were observed (*F*_2,14_ = 0.83, *p* = 0.43; **Figure 4C**).

## Discussion

### SCP-Induced Effects on the Spontaneous Spatial Recognition Memory

Our results showed that the nonselective muscarinic ACh receptor antagonist SCP impaired the marmosets’ ability to detect changes in the spatial location of ethologically irrelevant stimuli in the environment. When assessed on the murine-based one-trial SOL recognition task (Ennaceur et al., 1997), SAL-treated animals explored the displaced object significantly longer than its identical stationary counterpart (i.e., exploration time and discrimination ratio). Captive callitrichids seem to readily respond to environmental change, particularly when spatial cues are involved (Gaudio and Snowdon, 2008) – an aspect possibly related to their use of highly seasonal habitats (Stevenson and Rylands, 1988). The exploratory preference for the displaced object in this group seems unlikely to be due to changes in object-related motivation or perception, or even overall activity, as total exploration and locomotor activity remained unaltered between the sample and test trials.

On the other hand, SCP-treated animals explored both objects equivalently during the test trial. To the best of our knowledge, NHPs have not yet been assessed in SOL tasks. The performance of rodents, however, is generally impaired following both systemic administrations (Murai et al., 2007; Pitsikas, 2007; Schäble et al., 2012) and local infusions of SCP into the perirhinal and medial prefrontal cortices (Barker and Warburton, 2009), as well as after selective immunotoxic lesions of central cholinergic systems (medial septum/vertical limb of the diagonal band; Easton et al., 2011). It is important to note as well that the SCP-treated marmosets explored both objects as much as the SAL group explored the displaced item. This was also the case when rodents were systemically administered the same antagonist (Schäble et al., 2012). As SCP was given 20 min prior to the sample trial, this treatment may have impaired the acquisition of relevant task-related information and thereby this group later perceived both objects as being novel rather than familiar. In rats, muscarinic blockade impaired the initial encoding phase of SOL, whilst sparing information retrieval (Barker and Warburton, 2009). This phase-dependent effect was also consistently shown in both rodents (e.g., Barker and Warburton, 2009) and NHPs (e.g., Aigner et al., 1991b) assessed in spontaneous and reinforced visual recognition tasks, respectively, as well as in healthy human volunteers (e.g., Atri et al., 2004). However, McTighe et al. (2010) found that direct damage to the perirhinal cortex led rodents to treat novel objects as familiar stimuli. Therefore, different factors may influence recognition memory processes, including the specific brain area involved, neurochemical mediator, task demands and animal model. Alternatively, the current SCP treatment may have impaired attentional and/or perceptual processes that are also required for the task (Voytko, 1996), yet this does not seem to be the case in our marmosets. Total object exploration remained constant from the sample to the test trial, with no significant between-group differences being observed as well. Furthermore, we did not observe drug-induced changes in general locomotor activity. Although SCP has been shown to induce hyperactivity (Day et al., 1991), others reported a decrease (Besheer et al., 2001) or even a lack of effect (Schäble et al., 2012), leading to the suggestion that methodological aspects contribute significantly to the observed outcome (e.g., dose, behavioral task, administration route, gender; Klinkenberg and Blokland, 2010).

With the growing use of the several close variants of the spontaneous recognition memory task in rodents (Ameen-Ali et al., 2015), our results may have important prospective implications for the development of a preclinical cross-species procedure to assess specific memory functions. However, more comprehensive studies in marmosets are warranted, given that in rodents, for example, not all types of spontaneous recognition memories are affected by central cholinergic activity. Rats with selective immunotoxic lesions of specific basal forebrain cholinergic projections to the hippocampus were unable to recognize simple spatial representations (Easton et al., 2011), although other types of spatial memory tasks and more complex episodic-like memories remained intact with the lack of basal ACh input to the hippocampus or temporal/frontal cortex (Baxter et al., 1995; Easton et al., 2011). In marmosets, immunotoxic lesions of specific ACh neurons within the basal forebrain (Ridley et al., 1999) caused similar mnemonic deficits as those induced by ablation/excitotoxic damage to their respective target areas (Ridley et al., 1995; Barefoot et al., 2002) or even by SCP treatment (Harder et al., 1998), thus indicating that it may act more in terms of maintaining the proper functionality of their projection sites. If so, this rising signaling pathway probably affects long-term information encoding of different memory types, depending on its target structure (Harder et al., 1998). It is important to note that significant differences between rats and NHPs have been reported in terms of the cytochemical organization of basal forebrain cholinergic neurons, although the latter corresponded to that of humans (Geula et al., 1993). Therefore, it would be interesting to evaluate the effects of specific lesions on spontaneous recognition tasks in marmosets, as only visual discrimination and conditional learning tasks have been assessed. Pharmacological blockade of muscarinic receptors has consistently resulted in deficits in visual discrimination tasks of DNMS (reviewed in Robinson et al., 2011), similar to our current results in a spontaneous spatial recognition task. M1 receptor agonism was shown to enhance the cognitive performance and/or reverse SCP-induced deficits in these tasks (Rupniak et al., 1989; Carey et al., 1992; Harries et al., 1998; Lange et al., 2015).

### SCP-Induced Effects on FMBs

We also demonstrated that the SCP-induced blockade of cholinergic neurotransmission disrupted the marmosets’ ability to associate a predator-related stimulus with the specific spatial context in which it was encountered. On one hand, after a single brief encounter with the aversive stimulus, SAL-treated animals spent significantly less time in the specific snake-paired section of the OF arena, but more time being vigilant of their surroundings compared to pre-exposure levels. Concurrent changes in general activity were not observed. Snakes prey on marmosets (Teixeira et al., 2016), and as a result even inanimate related stimuli elicit a fear response in both feral and captive individuals (e.g., Barros et al., 2002). Le et al. (2013) have even argued that snakes exerted a prominent role in the development of primate neural structures, with minimal (Mineka et al., 1984) or no prior contact (Vitale et al., 1991) leading to persistently strong fearful reactions in NHPs. Exactly as we recorded in our subjects, during an encounter feral marmosets typically emit *tsik–tsik* alarm calls and visually inspect the snake; they never freeze (Ferrari and Lopes Ferrari, 1990; Teixeira et al., 2016). However, after the event, they act cautiously and avoid the interaction site for up to several days (Bartecki and Heymann, 1987). This indicates that: (1) our marmosets perceived the snake model as an unconditioned threat (e.g., Clara et al., 2008); (2) their post-encounter hypervigilance in the training context may be akin to the behavioral response of rodents during contextual fear-conditioning procedures using footshocks (*freezing*: reviewed in Maren et al., 2013) or predators (*risk assessment*: Ribeiro-Barbosa et al., 2005); and (3) subsequent avoidance of this specific location seems to be in line with the fear-induced conditioned-place-aversion (CPA) response seen in rodents (e.g., Zanoveli et al., 2007) and in NHPs under natural settings (Bartecki and Heymann, 1987; van Schaik and Mitrasetia, 1990; Isbell and Etting, 2017). Neurobiological studies on FMBs in NHPs is mostly focused on their unconditioned reaction to explicit aversive stimuli (e.g., predator, conspecifics), yet fear learning has been experimentally assessed using different paradigms, such as fear-potentiated startle (e.g., Antoniadis et al., 2009), cue-conditioning (Agustín-Pavón et al., 2012), observational conditioning (e.g., Mineka et al., 1984) and passive avoidance (e.g., Machado et al., 2009).

On the other hand, in the SCP-treated group, post-exposure vigilance and dwell time in the snake-paired section of the apparatus did not differ from the initial baseline levels of either group. This seems unlikely to be due to a drug-induced effect on their visual perception or behavioral response to the snake model. During their encounter with this stimulus we recorded similar levels of visual inspection and *tsik–tsik* alarm calls in both groups. The SCP group did, nonetheless, spend more time in motion than the SAL-treated animals. Although SCP may induce hyperactivity, as mentioned above, the difference was already present on the initial pre-exposure trial and as such may be a drug-unrelated feature inherent to that group.

The role of cholinergic signaling in fear learning of NHPs has yet to be fully addressed. Nonetheless, results from our present study seem to indicate that muscarinic antagonism may disrupt the encoding of conditioned fear responses for a spatial context in marmosets. In rodents, systemic and intra-hippocampal infusions of SCP selectively impaired the acquisition of a conditioned freezing response for the training context previously paired with an aversive footshock (recently reviewed in Wilson and Fadel, 2017). Selective antagonism of muscarinic M1 receptors (Soares et al., 2006) and pre-training electrolytic lesions of central cholinergic projections to the hippocampus yielded similar results (Maren and Fanselow, 1997). Cholinergic blockade also disrupts fear learning measured in other behavioral tasks in rats (e.g., inhibitory avoidance; reviewed in Robinson et al., 2011). However, the role of muscarinic signaling on the retrieval of aversively motivated behavior is still unclear (reviewed in Robinson et al., 2011; Wilson and Fadel, 2017).

## Conclusion

Our results indicate that the pharmacological blockade of cholinergic neurotransmission with SCP impaired the marmosets’ ability to detect changes associated with the spatial location of ethologically irrelevant (SOL task) and relevant stimuli (FMB task). However, at present, we are only able to argue that cholinergic deficiency affects the way SOL recognition and aversive learning are processed in the short-term. Further studies are required to properly ascribe the role of ACh on the different phases of the information processing systems, their related brain circuits and the specific resultant effects. Similar investigations using longer retention intervals (>10 min), distinct objects/cues and gender comparisons will also contribute with important complementary information to our current understanding on normal and dysfunctional learning and memory processing in NHPs and potentially in humans. This novel approach, using a spontaneous (spatial) recognition task, may prove useful in terms of providing a means for a direct cross-species comparison between NHPs and rodents. Compared to other simians, the marmosets’ small body size, rapid reproductive turnover, shorter life-span, high adaptability to captivity and lower husbandry costs (reviewed in Barros and Tomaz, 2002), while still retaining a high anatomical and neurochemical resemblance to their larger counterparts (Stephan et al., 1980; Geula et al., 1993), makes them a unique model for human neuropathologies. Marmosets may thus help reduce the translational gap between pre-clinical studies and memory-associated human pathologies.

## Author Contributions

JM, MB: conception and design; acquisition, analysis, and interpretation of data; drafting the article and revising it critically for important intellectual content. FdJ: conception and design; acquisition, analysis, and interpretation of data. RM: drafting the article and revising it critically for important intellectual content.

## Conflict of Interest Statement

The authors declare that the research was conducted in the absence of any commercial or financial relationships that could be construed as a potential conflict of interest.
